# Distributed Humidity Sensing in PMMA Optical Fibers at 500 nm and 650 nm Wavelengths

**DOI:** 10.3390/s17040738

**Published:** 2017-03-31

**Authors:** Sascha Liehr, Mathias Breithaupt, Katerina Krebber

**Affiliations:** Bundesanstalt für Materialforschung und–prüfung (BAM), Unter den Eichen 87, 12205 Berlin, Germany; mathias.breithaupt@bam.de (M.B.); katerina.krebber@bam.de (K.K.)

**Keywords:** optical fiber sensor, humidity sensor, distributed sensor, OTDR, polymer optical fiber, backscatter measurement, Rayleigh scattering, PMMA, spectral measurement

## Abstract

Distributed measurement of humidity is a sought-after capability for various fields of application, especially in the civil engineering and structural health monitoring sectors. This article presents a method for distributed humidity sensing along polymethyl methacrylate (PMMA) polymer optical fibers (POFs) by analyzing wavelength-dependent Rayleigh backscattering and attenuation characteristics at 500 nm and 650 nm wavelengths. Spatially resolved humidity sensing is obtained from backscatter traces of a dual-wavelength optical time domain reflectometer (OTDR). Backscatter dependence, attenuation dependence as well as the fiber length change are characterized as functions of relative humidity. Cross-sensitivity effects are discussed and quantified. The evaluation of the humidity-dependent backscatter effects at the two wavelength measurements allows for distributed and unambiguous measurement of relative humidity. The technique can be readily employed with low-cost standard polymer optical fibers and commercial OTDR devices.

## 1. Introduction

The measurement of humidity or moisture is an important task in various fields of applications, for example in industrial process control, agriculture, domestic applications, air condition control, or the civil engineering and structural health monitoring sectors. Especially for the monitoring of extended structures and water ingress detection, a distributed (spatially resolved) measurement is important. This cannot be adequately achieved with conventional humidity point sensors. 

Common principles for the measurement of humidity are summarized in [1,2]. Apart from electrical and mechanical effects, more and more optical techniques have been proposed and are being used. Although not yet commercially significant, fiber optic techniques have been identified to have various advantages over conventional techniques [3,4,5]. Fiber optic humidity sensors are small, immune to electromagnetic interference, and can be multiplexed, operated remotely and in situ materials and structures. Various fiber optic humidity sensing techniques have been proposed, for example based on absorption loss measurement [6], evanescent wave interaction [7,8] or fiber Bragg gratings in silica fibers [9,10,11] and polymer fibers [12,13,14]. Most fiber optic principles have the potential to be multiplexed and act as point sensors or sectional sensors. One way of spatial multiplexing can be achieved by using the optical time domain reflectometry (OTDR) technique. The majority of OTDR-based humidity sensor implementations only allow for quasi-distributed measurement, for example involving induced macrobend loss [15,16] or recoating of silica fiber sections with a humidity-sensitive film [6]. Michie et al. proposed a distributed water detection sensor design based on a water-swellable polymer provoking microbend optical loss along a silica optical fiber [17]. 

The approach presented in this article has the advantage of true distributed (continuously and spatially resolved) humidity measurement using intrinsic scattering and absorption effects in polymer optical fibers (POFs). Distributed measurement is achieved by analyzing backscattering and attenuation parameters along the fiber. We previously proposed distributed relative humidity (RH) measurement in standard poly(methyl methacrylate) (PMMA) POFs at 650 nm wavelength [18,19]: backscatter traces showed localized RH-dependent backscatter changes and transmission loss changes. A similar RH-dependent backscatter change effect, but at impractically small magnitude, has also been observed in low-loss perfluorinated POFs based on the CYTOP™ polymer [20] using incoherent optical frequency domain reflectometry (I-OFDR) [21]. The humidity-dependent characteristics (Rayleigh backscatter decrease and reduced transmission with increasing RH) in PMMA POFs [18,19,22,23] have been initially investigated as cross-sensitivity effects when characterizing the Rayleigh backscatter dependence for distributed strain sensing and structural health monitoring applications [24,25,26]. POF has the unique advantage to endure and measure strain of more than 40% [24] and exceeding even 100% [20,27]. 

In this article, we present the detailed results of Rayleigh backscattered power dependencies and transmission dependencies on RH changes at 500 nm and 650 nm wavelengths. The combination of these two effects and their differing characteristics at different wavelength measurements (using a dual-wavelength OTDR) are analyzed for unambiguous identification and localization of RH changes and to distinguish other backscatter-dependent effects.

## 2. Materials and Methods

All measurements have been conducted with standard step-index PMMA POFs (Mitsubishi’s Eska GK-40) with a 980 µm diameter PMMA core, a 10 µm thin fluorinated polymer cladding and a numerical aperture of NA = 0.5. The OTDR technique provides spatially resolved backscatter measurement analogous to the radar technique: short optical pulses are sent into the fiber and backscattered power is sampled as a function of time or distance. The OTDR measurements have been performed with a photon counting OTDR (*υ*-OTDR by Luciol Instruments) with a sampling resolution of 2.6 cm and a spatial resolution of about 10 cm. The pulse optical wavelength of the OTDR can be switched between 500 nm and 650 nm, the favorable optical transmission windows for PMMA POF. The photon counting technique provides very high sensitivity and spatial resolution [28]. However, the spatial resolution using the investigated high-NA step-index fiber type is limited by the modal dispersion along the fiber and decreases to about 2 m at 90 m distance. This effect could be reduced by using lower NA fibers or gradient-index PMMA-based POFs. Experiments with these fiber types are pending. 

**Figure 1:**
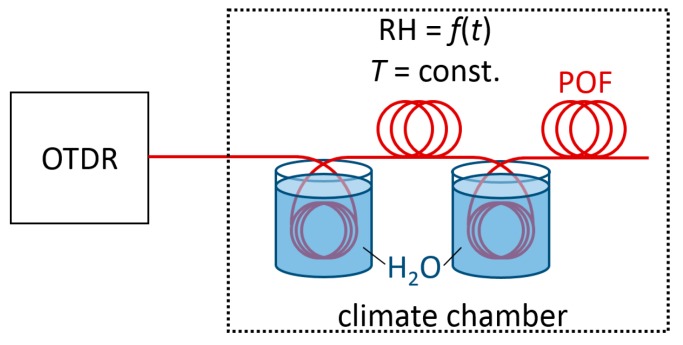
Schematic of the measurement setup.

Our aim was to investigate all backscatter effects at different optical wavelengths (in this case at 500 nm and 650 nm) and analyze their differing humidity-dependent characteristics for distributed RH sensing. Since the observed response times of the humidity-dependent backscatter changes are relatively slow, the integration time for each backscatter measurement has been chosen to be 1 h. OTDR traces at both wavelengths have been recorded alternatingly at constant temperature and varying RH settings. All OTDR measurements have been conducted with the setup depicted in Figure 1: the POF has been placed in a climate chamber at a constant temperature and variable RH with two fiber sections placed in distilled water (distance *l* ≈ 21–37 m and *l* ≈ 58–69 m). The POF sections in the water basins have a saturated water content and act as a reference during the RH cycles presented in Section 3.

PMMA can take up to about 2% water by mass. Investigations on different PMMA materials show that the rate of water sorption increases with increasing temperature but the equilibrium concentration of water does not depend on the temperature [29]. We also expect this behavior for PMMA step-index POFs. All measurements have been conducted at a constant temperature of *T* = 30 °C.

## 3. Results

In this section, the measured RH-dependent effects of spectral transmission change, transmission change at the OTDR wavelengths, backscattered power change and detected fiber length change are presented. Each effect is briefly discussed at the end of the respective sub-section. A more general discussion of the POF RH sensor characteristics and the impact of cross-sensitivities is given in Section 4.

### 3.1. Spectral Transmission Dependence on Relative Humidity

**Figure 2:**
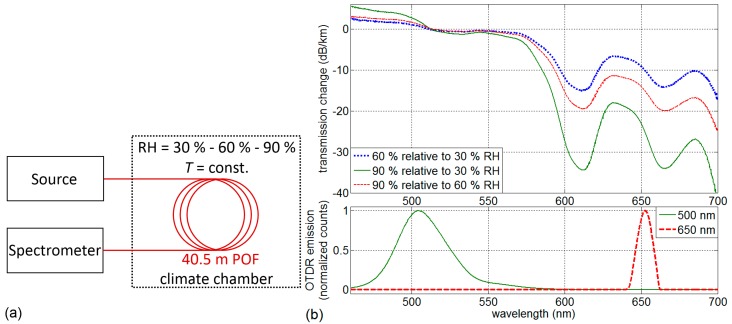
(**a**) Schematic of the transmission change measurement setup; (**b**) spectral transmission changes of PMMA POF (upper plot) and measured OTDR emission spectra (lower plot).

In order to verify the measured OTDR transmission dependence and identify favorable wavelength measurements for future sensor designs, spectral transmission changes have been conducted. Figure 2a shows the schematic of the setup comprising a halogen source and a high resolution spectrometer (Ocean Optics HR4000) that has been used for optical transmission change measurements of a *l_f_* = 40.5 m long POF. Three transmission measurements have been conducted during saturated RH values of 30%, 60% and 90%. Figure 2b shows the logarithmic plots of the measured spectral transmission relations (or changes) between different saturated RH settings: 90% RH transmission spectrum relative to 30% RH transmission spectrum, 60% RH relative to 30% RH and 90% relative to 60% RH, respectively. The emission spectra of the OTDR device (LED at 500 nm and laser diode at 650 nm) are shown in the lower plot of Figure 2b.

The RH-dependent transmission change results in PMMA POF show increasing transmission loss with increasing RH for wavelengths exceeding about 513 nm with distinct absorption peaks at 612 nm and 665 nm. The cause for the transmission changes are impacts of the OH vibrational absorptions, presumably 5*υ*_OH_ and 4*υ*_OH_ + δ_OH_ [30,31], and their influence on CH vibrational absorptions at 622 nm and 670 nm. In contrast, the wavelength range below 513 nm exhibits increasing transmission with increasing RH. The transmission change measurements show that the wavelength range of 500–550 nm is favorable in terms of transmission stability. However, the relatively weak but measurable transmission change at 650 nm can actually be used for distributed humidity sensing by deploying distributed backscatter measurement techniques. The OTDR emission spectra peaks are around 505 nm and 653 nm. The OTDR-specific transmission changes Δ*P_a/b,OTDR_* at the two wavelengths are calculated from the measured transmission spectra (counts) of *P_RH,a_(λ)* at RH *a* relative to *P_RH,b_(λ)* at RH *b* and the actual OTDR source spectra *P_OTDR_(λ)* at 500 nm and 650 nm (*P*_500*nm*_*(λ)* and *P*_650*nm*_*(λ)*, respectively) as generalized in this equation:

(1) $$\Delta P_{a/b,OTDR}=10\log_{10}\left[\sum_{x\in \lambda }\frac{P_{RH,a}\left(x\right)}{P_{RH,b}\left(x\right)}\;\frac{P_{OTDR}\left(x\right)}{\sum_{y\in \lambda }P_{OTDR}\left(y\right)}\right]\frac{1000\;m}{l_{f}}$$

The transmission changes Δ*P*, calculated from measured transmission and emission spectra, are summarized in Table 1 for saturated RH values at 30%, 60% and 90%. 

As expected from Figure 2b, transmission changes at 500 nm exhibit small positive values (transmission increase with increasing humidity). Regarding the optimum emission wavelengths for unambiguous bi-spectral humidity measurement (RH-dependence of one wavelength and a second wavelength independent of RH), the wavelengths around 500 nm and 650 nm are favorable. In addition to the availability of low-cost optical components for these wavelengths, they also have the advantage of falling into the minimum attenuation windows of PMMA. The transmission change impact is studied in more detail in the following section.

### 3.2. Distributed Backscatter and Transmission Dependence

**Figure 3:**
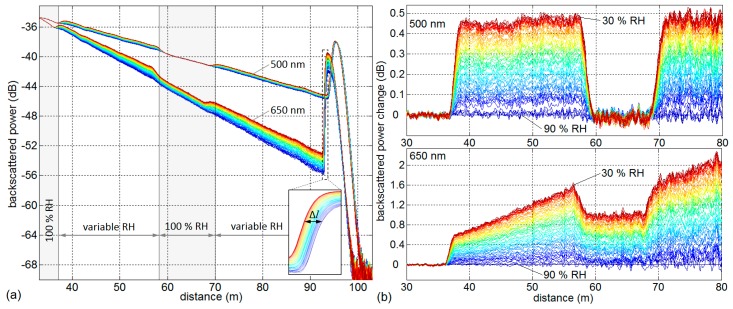
Multiple OTDR backscatter results at 500 nm and 650 nm during decreasing RH from 90% to 30% (from blue to red, compare Figure 5 and Figure 6: data during the time period *t* = 17 d to *t* = 22 d): (**a**) OTDR backscatter traces and (**b**) backscattered power changes relative to *l* = 35 m and 90% RH.

Distributed backscatter change and transmission change is obtained from relative backscattered power evaluation of OTDR measurements. Figure 3 shows OTDR backscatter traces (a) and backscatter change (b) relative to a reference trace at 90% RH for both wavelengths. Due to the lower attenuation at 500 nm compared to 650 nm (about 90 dB/km compared to 151 dB/km in one-way transmission at 30% RH), the results obtained at 500 nm exhibit a higher signal-to-noise ratio. The POF sections with constant saturated RH in the water basins are clearly visible at *l* ≈ 21–37 m and *l* ≈ 58–69 m and are used as a reference power level for relative backscatter change evaluation.

Two effects are evident at 650 nm: increased backscattered power level and superimposed transmission loss related to increased water content in the fiber at higher RH settings. As visible in the spectral transmission results in Figure 2, the transmission change at 500 nm is negligible (compare between *l* = 35 m and *l* = 65 m). Only the backscattered power change can be observed at *l* = 37–58 m and *l* > 69 m. 

**Figure 4:**
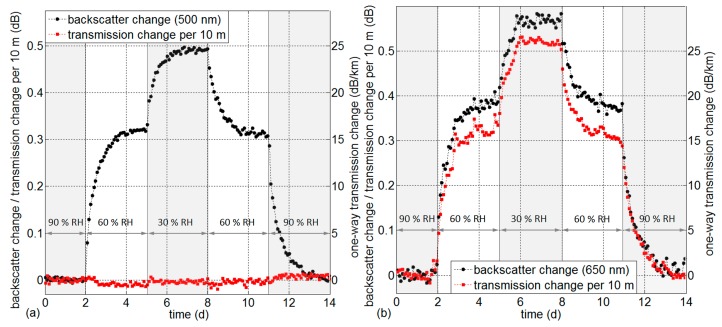
Backscatter change and transmission change during step-wise RH changes for (**a**) 500 nm and (**b**) 650 nm.

All following calculations of backscatter change and transmission change are conducted by comparing OTDR traces relative to a reference OTDR trace at a certain RH setting. The averaged backscattered power level of a fiber section subjected to varying relative humidity is compared to the averaged power level of a saturated fiber section in the water basin. The calculation of backscatter changes at 650 nm has additionally been adjusted by proportionally subtracting the measured optical loss changes over the compared fiber sections. It has to be noted that all relative change values are defined as change of backscattered power as it is detected by the OTDR. That means that transmission change over a given fiber length constitutes optical power loss that is accumulated by passing the fiber section twice: as forward-propagating pulse and as backscattered power. Transmission measurement readings at the end of the fiber would show half of that value for changes over the same fiber length. This definition corresponds to twice the “transmitted intensity” changes and “scatter intensity” changes presented in [18,19]. Figure 4 shows the two sensor effects at both wavelengths during a step-wise change of RH between 90% and 30% with an equivalent one-way transmission change in dB/km as a second *y*-axis for direct comparison with the spectral transmission results in Figure 2.

The results show reproducible and reversible characteristics. As indicated in Figure 3 (distributed backscatter changes), the increased water content in the POF induces backscatter changes at both wavelengths with a slightly smaller magnitude at 500 nm. The measured transmission change agrees very well in magnitude with the spectral transmission change measurement presented in Figure 2. It is negligible at 500 nm and corresponds to about 25.8 dB/km at 650 nm at 30% RH relative to 90% RH (comparable to 25.4 dB/km from spectral transmission results in Table 1). Both effects, backscatter and transmission change, exhibit similar response times and asymptotically approach an equilibrium after about 1.5 days. The response time to 50% of the saturated value is about 6 h and the response time to 90% of the saturated value is about 31 h. The possibility of reducing the response time by using fibers of a smaller diameter is discussed in Section 4. The 10 µm fluorinated cladding does not seem to delay the water uptake into the PMMA core. It has been shown that even perfluorinated core fibers based on CYTOP absorb water with a response time of a few hours at 70 °C [20].

**Figure 5:**
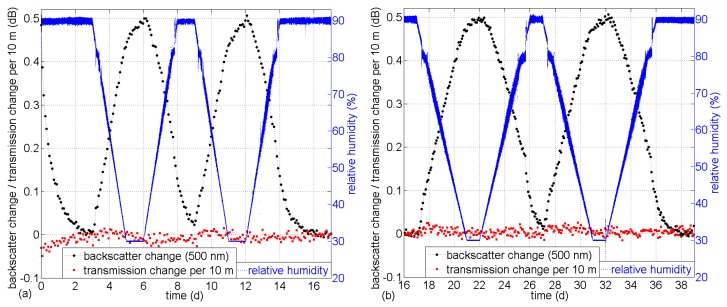
Backscatter change and transmission change during gradual RH changes at 500 nm wavelength (**a**) for slope durations of 2 days and (**b**), continued, for slope durations of 4 days.

**Figure 6:**
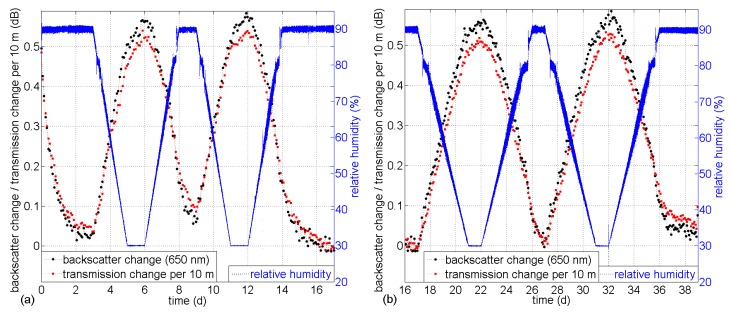
Backscatter change and transmission change during gradual RH changes at 650 nm wavelength (**a**) for slope durations of 2 days and (**b**), continued, for slope durations of 4 days.

The same backscatter and transmission change evaluation as above has been conducted during gradual RH change cycles between 30%and 90% while maintaining constant RH for 1 day at the reversal values. The resulting backscatter change and transmission change characteristics are shown for slopes of 2 days (a) and 4 days (b) at 500 nm in Figure 5 and at 650 nm in Figure 6. 

**Figure 7:**
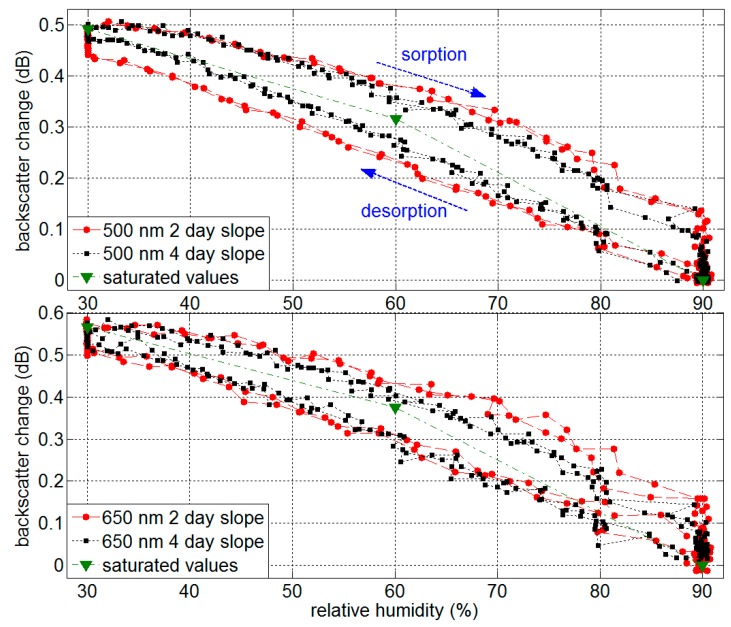
Backscatter change during linear RH changes (2-day and 4-day slope for two full cycles) and saturated RH values at 500 nm and 650 nm (the sorption/desorption deviation is due to the response time of the backscatter change effect).

The results show good reproducibility and reversibility and agree well in magnitude with the results obtained during step-wise humidity changes between 30% and 90% RH in Figure 4. Figure 7 shows the backscatter changes during two full cycles (compare data in Figure 5 and Figure 6 for slope durations of 2 days and 4 days) as a function of RH in the climate chamber. Also the backscatter change values for saturated RH from Figure 4 are added to the plot. 

The apparent “hysteresis” is due to the response time of the sensor and is less distinct for lower RH gradients during the 4-day slope. Saturated water content in the fiber shows reproducible results without hysteresis, as presented in Figure 4. The buckling at 81% RH is due to the discontinuity of the RH gradient in the climate chamber (compare RH trace in Figure 5). 

Significant response time differences between sorption and desorption are not visible in Figure 4 and Figure 7, although the diffusion coefficients for PMMA have been determined to be 6.4 × 10^−9^ cm^2^s^−1^ and 9 × 10^−9^ cm^2^s^−1^ for sorption and desorption [32], respectively. Also, Zhang et al. [14] reported deviating results of POF fiber Bragg grating sorption/desorption response times. Mechanisms of sorption and desorption have been discussed by Turner [32]. It is assumed that the sorption of water is partly due to dissolution in the polymer network, causing a swelling of the fiber (discussed in Section 3.3). The remaining part is accommodated in microvoids in the polymer. The relation of these effects (dissolved water/total water uptake) is in the order of 0.7 [33]. 

We assume that the Rayleigh backscattered power dependence may be partly caused by the reduction of refractive index differences at the interfaces between the polymer network and the microvoids as well as within the polymer structure itself due to water uptake and the filling of gaseous voids by water molecules. This may result in a reduced inhomogeneity of the refractive index along the fiber and, as a consequence, decreased scattering. It can also be speculated that the humidity-induced swelling of the polymer causes a mechanically forced closing of the microvoids and micro-crazes which in turn leads to an increase of material homogeneity and, therefore, reduced scattering. The exact reasons, however, are unknown. The response time is further discussed in Section 4.

### 3.3. Impact on Measured Length Change

**Figure 8:**
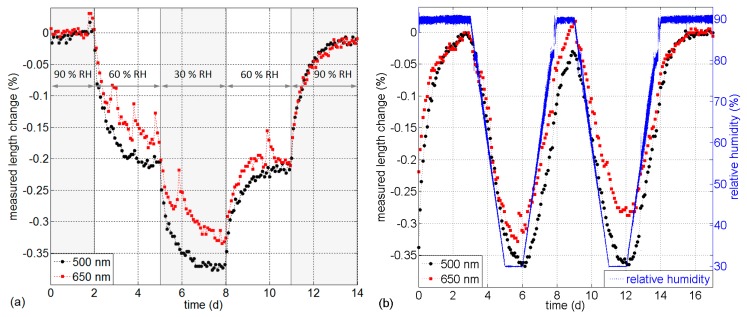
Measured length changes in % for 500 nm and 650 nm during (**a**) step-wise RH change and (**b**) gradual RH change.

The absorption of water into the fiber has also an effect on the optical runtime of the light. Lower RH values result in decreased runtime, meaning a reduced measured fiber length. This is visible in the shift of the fiber end reflection peak (see Figure 1a). The length change Δ*l*, normalized to the fiber length that is exposed to RH changes, is obtained from the interpolated shift of the rising slope of the fiber end reflection peak (at 3 dB below peak value). The measured length changes during step-wise RH changes (a) and gradual changes (b) are shown in Figure 8. These results have been obtained from the same OTDR measurements and experiments presented above.

The measurements indicate reduced “fiber length” at lower RH values and a response time comparable to that of the backscattered power change and transmission change effects in Figure 4. The increased optical runtime in the POF with increasing water content can be mainly attributed to the swelling of PMMA due to water uptake, which is in the order of 0.4% [29]. This swelling effect is nominally (about 40 × 10^−6^ (%RH)^−1^) more significant than the opposite impact of the RH-dependent refractive index change of about Δ*n* ≈ −11 × 10^−6^ (%RH)^−1^ [34,35]. Measurements of the temperature-dependence of the refractive index change as a function of RH for deuterated PMMA showed a strong temperature dependence Δ*n*/ΔRH = *f*(*T*) and a change of sign (from positive to negative) at temperatures exceeding about 60 °C [36]. This indicates that Δ*n*/ΔRH for PMMA may also exhibit a significant temperature dependence.

If the measured fiber length change is intended to be used for humidity measurement, temperature variations of the sensor also have to be considered: besides the unknown Δ*n*/ΔRH dependence of PMMA, the temperature-dependent refractive index change of PMMA is negative with about −137 × 10^−6^ K^−1^ at 20.1 °C [37]. The linear thermal expansion coefficient is in the order of 75 × 10^−6^ K^−1^ [29] and also depends on the water content in the fiber ranging from 70.4 × 10^−6^ K^−1^ to 92.8 × 10^−6^ K^−1^ at 50 °C [33]. We determined the effective length change of the investigated GK-40 POF to be relatively linear at about −54 × 10^−6^ K^−1^ [25]. Depending on the sensor fiber integration (loosely integrated with overlength or bonded to the structure), the expansion coefficient of the surrounding material also has to be considered. It is important to note that the mechanical impact on the fiber (not necessarily strain) can have a considerable impact on the detected length changes. Reasons for this are the strong modal dispersion in this step-index fiber and the mechanically induced redistribution of modal powers and propagation mode filter effects. Since the fiber length change only provides information about the entire fiber, it is omitted from the discussion of distributed effects, cross-sensitivities and applications.

## 4. Discussion

The aim of this article is to identify the suitability of PMMA POF for distributed humidity measurement in general, and at different wavelengths in particular. The results are very promising. The resolution of the RH changes depends on the signal-to-noise ratio of the OTDR trace, i.e., the fiber distance, the OTDR integration time and the length of the evaluated fiber section. For a saturated condition, a resolution in the order of 1–2% RH could be achieved. The following cross-sensitivities and limitations have to be considered in an application scenario. 

A relevant cross-sensitivity is the fiber’s backscatter dependence on ***temperature***. We already investigated this effect for PMMA POF [25,38] and low-loss perfluorinated POF (CYTOP) [20,24]. Temperature change in PMMA POF also results in a relatively linear change of the backscattered power level of about 0.01 dB/K but does not cause measurable transmission change. This behavior could be mistaken for RH changes if only 500 nm backscatter traces are evaluated. A temperature difference of 10 K would translate to a RH change of about 12% (assuming 0.5 dB linear change for RH change between 30% and 90%). However, in combination with the additional transmission change effect at 650 nm, temperature changes and RH changes along the fiber can be distinguished and unambiguously identified. For many applications, for example in the geotechnical field or building foundations, the temperature is relatively constant and its impact may be neglected.

It has to be noted that also ***strained fiber*** sections cause increased backscatter levels [24,25]. Therefore, it has to be assured that the sensor fiber is installed “strain-free” and possibly with overlength for applications where significant strain can be expected. As it is the case for temperature, strain induces negligible attenuation. A combined evaluation at 500 nm and 650 nm could distinguish strain and humidity impact.

The ***response time*** of the sensor is slower than that of most other humidity sensors. The field of application is thus limited to slowly varying RH changes or applications where the immediate response to humidity change is not crucial. However, the response time can be improved to a few hours or less by using commercially available standard PMMA fibers with smaller core diameters, for example 500 µm or 250 µm diameters. Response times below one hour may be possible with a 250 µm PMMA fiber. For comparison: a response time of 30 min has been reported for a FBG in a 200 µm PMMA fiber [13]. Further reduction of the fiber diameter to 135 µm reduced the response time to 12 min [14] and even down to a few seconds in a 25 µm diameter fiber [39]. Also, fibers with various polymer jacketing materials are available with the advantage of chemical or mechanical impact protection. We already showed that such more rugged POF fibers with polyethylene or polyacrylate jacketing materials also respond to humidity, but at increased response times [18].

The RH measurement is based on the evaluation of backscattered power changes relative to a ***reference*** ***measurement*** or relative to a ***reference fiber section*** with a known RH or water content. We used water-saturated fiber sections as a reference. If such a reference condition cannot be provided, only relative changes along the sensor fiber can be measured. This could be viable for applications where water ingress or moisture change has to be detected in an otherwise homogeneous structure. For direct RH measurement, however, one or more reference sections are necessary. Such a reference backscatter power level may also be provided by inscribing constant scattering damage structures into the POF core using focused femtosecond laser pulses [38]. In this way, absolute temperature measurement has been achieved in PMMA POF by relating constant scattering damage power to temperature-dependent Rayleigh scattering power changes. 

The advantage of the approach presented in this article is the distributed measurement capability over an extended sensor length of up to 200 m. The technique has the potential for humidity or moisture measurement not only in gases but also in solid state materials and structures. Due to the distributed measurement along a flexible sensor fiber, continuous and spatially resolved humidity measurement can be conducted. Also humidity changes of fiber sections shorter than the spatial resolution could be detected since power changes are accumulated on the backscatter trace (the backscatter trace is a convolution of the optical pulse shape with the RH-induced backscatter change). The combined advantages of distributed and gapless measurement is beneficial in various fields of application.

***Application examples*** can be found in the structural health monitoring sector for humidity/moisture monitoring in building foundations, the measurement of moisture content in concrete and screed or moisture ingress detection in sealed structures such as bridges. Other applications that can benefit from distributed RH measurement are seepage line monitoring in dykes and dams or the detection of leaks in landfills, industrial facilities and reservoirs. In most of these applications, the humidity ingress mechanisms are either slow or the required response time to take action is not crucial. Moisture penetration, for example in buildings and foundations, is a very slow process with critical consequences on the long-term structural integrity or public health, for instance due to mold formation. The limited response time is not an issue for these applications. As mentioned above, decreasing the response time by using smaller diameter fibers would further extend the range of applications.

The distributed sensing capability is essential if local water ingress or leakage in extended structures is to be detected. These applications can often not be sufficiently serviced with a point sensor approach which may require the installation of hundreds of point sensors and the associated wiring effort. Distributed sensing, on the other hand, allows for precise localization of humidity changes as well as the spatial spreading of the humidity influx over time along a single fiber. Another application example is the distributed measurement of concrete curing and setting processes inside large concrete infrastructures. The information of the spatial humidity distribution inside the structure can be used to optimize the moisture content during curing as well as the construction progress.

The increased cost of an OTDR unit can be economical for applications where water ingress or leakage has to be monitored over extended distances and a great number of point sensors can be substituted. OTDR-based sensing could also be cost-efficient if the unit does not have to be installed permanently, for example during a temporary curing monitoring application, or when regular service interval measurements are sufficient. The fiber sensor itself is low-cost and can be installed in any orientation (linear or meandering) to monitor an area, volume or along extended structures. Sensor fibers can also be installed without the initial intent of monitoring the structure at close to zero cost, providing the potential for later activation for distributed humidity measurement. A reduction in cost can be further achieved in large-scale applications since multiple sensors can be multiplexed in a sensor network and silica fibers could be used as lead fibers to extend the attenuation-limited measurement distance of a POF RH sensor network.

## Figures and Tables

**Table 1:** Calculated transmission changes from measured OTDR spectra and transmission spectra.

RH Settings	Δ*P* at 500 nm	Δ*P* at 650 nm
60% relative to 30%	0.4 dB/km	−10.2 dB/km
90% relative to 30%	1.2 dB/km	−25.4 dB/km
90% relative to 60%	0.8 dB/km	−15.3 dB/km
